# Visitors flow management at Uffizi Gallery in Florence, Italy

**DOI:** 10.1007/s40558-022-00231-y

**Published:** 2022-07-28

**Authors:** Alessandro Attanasio, Maurizio Maravalle, Henry Muccini, Fabrizio Rossi, Gianluca Scatena, Francesco Tarquini

**Affiliations:** Department of Information Engineering, Computer Science and Mathematics, University of L’Aquila, Via Vetoio, L’Aquila, 67100 Italy

**Keywords:** Access management, Cultural tourism, Predictive analytics, Prescriptive analytics, Queue management system, Smart cities

## Abstract

We present a data-driven solution to manage visitors’ access at the Uffizi Gallery in Florence, Italy. The goal is to avoid the long lines outside the Museum, improving not only visitors’ experience, but also decency and security in the urban area. The solution implements a queue management system based on two data analytics models, one predictive and one prescriptive, which determine the entry time of each visitor. The system, which requires a minimal hardware and software infrastructure, was on the field from October 2018 to January 2020 during the most crowded visiting days, namely the free access days. First we report on the whole design and implementation process, then we show the solutions effectiveness and discuss the lesson learned.

## Introduction

The Uffizi Gallery in Florence is one of the most visited museum in Italy (ISTAT 2016) reaching over two millions visitors in 2018. Besides for its priceless masterpieces by Botticelli, Michelangelo, Raffello Sanzio da Urbino, Leonardo and many more, Uffizi is also known for potentially long waiting queues. The main reasons are:

- (i) the maximum number of people allowed to stay in the exhibition halls at any time of the day is fixed and limited;
- (ii) tourist presence is concentrated in certain days of the week and certain times of the year;
- (iii) the individual visiting time is not limited a-priori, and visitors are allowed to spend even the whole day in the Museum.

This paper reports on our experience in designing and implementing an access management solution that allows visitors to book *on-site* tickets for a visit *at a preferred available time during the day*, guaranteeing the chosen entry time *without queuing* outside the Museum. Our system computes the number of visitors allowed to access the Museum every 15 min. This figure is highly variable, and depends on both the number of visitors in the Museum and their actual visiting time. The main challenge consists in maximizing the number of accesses, while avoiding queues.

**Fig. 1:**
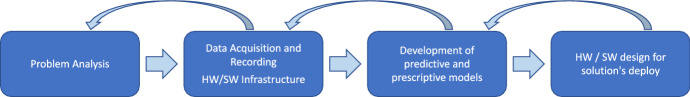
The process

The design process is summarized in Fig. 1. It is indeed highly iterative and incremental, with new questions arising from time to time, and new solutions being proposed incrementally.

Firstly, during the *Problem Analysis* phase, *we had to understand why huge queues form*. As described in Sect. 2, we gathered information from different stakeholders and designed a possible solution. Secondly, we had *to understand which data had to be collected to properly model the problem* and *to design and realize the necessary hardware and software infrastructure to collect and store the data*. This phase is described in Sect. 3, where we present the design of a simple but effective hw/sw infrastructure, an unprecedented facility for the Uffizi. Thirdly, we had to *explore the data to create predictive models* that could both forecast the number of visitors with sufficient accuracy and granularity, and to estimate the expected visit duration. These models are discussed in Sect. 4. Fourthly, we had to *use a prescriptive model to distribute visitors over the day* so as to maximize the number of visitors and reduce their waiting time. This is an *optimization* model presented in Sect. 5. Section 6 presents details of the hardware and software solution provided to both end-users and visitors. Finally, in Sects. 7 and 8 we discuss the lessons learned and outline future work.

## Problem description

Classical (conflicting) objectives of museums managers are: preserving the artworks, increasing the visitors number and improving visitors experience (Falk 2009). In this paper we focus on the initial phase of the visitor experience, that is, the time spent between accessing the Museum ticket office without a reservation and the actual entry time. Currently, visitors spend this time in a long line near the Uffizi entrance for various reasons. The most obvious is that the Museum has a limited capacity and cannot accommodate more than a certain number of people at the same time. Precisely, the safety policy requires that once the maximum capacity is reached, no one can enter the Museum until someone else leaves. The second reason is that neither is the duration of the visit limited, nor are visitors solicited to exit. Therefore, whenever the Museum is full up and the visitors in waiting line outnumber the leaving visitors, the outside queue starts growing. Additional factors increase the length of the waiting line:

- (i) a great number of tourists come to visit Florence and the Uffizi Gallery for one day only and with a peak in high season and/or on certain days of the week;
- (ii) a number of free access days (usually Sundays) have been introduced by the Ministry of Culture since 2017, resulting in a peak of visits with queues lasting more than 4 h;
- (iii) on weekdays, two separate queues exist: one priority line for visitors with a reservation, and a low priority line for those without a reservation. When the priority line grows, the low priority line may freeze.

Long lines surrounding the Museum area are a recurrent challenge of *overtourism* as they negatively affect local residents and urban decor (Pechlaner et al. 2019; Egresi 2018). Therefore, reducing or even eliminating these lines would be another relevant goal of an effective visitor management policy.

Visitors flow management, in famous and large museums, involves various stakeholders. In our case, the parties involved are the Director of the Uffizi Gallery, the Concessionaire responsible for ticketing and welcome services, the external Tour Operators and, last but not least, the visitors themselves. All those different views make a radical change in visitor management policies impossible: in fact, the Museum cannot easily increase its capacity and, at the same time, does not want to restrict access time or limit visit duration. Moreover, the current ticketing system and, in particular, the possibility of accessing the Museum without booking in advance must be preserved.

In this context, we designed a system that *virtualizes* the line, thus allowing visitors to enjoy other (not so well known) locations in Florence (Palazzo Pitti, Giardino di Boboli, Museo Archeologico, just to name a few) and/or the city’s surroundings, without overcrowding the Museum area or missing their turn in the queue. In detail, we conceived the following route for visitors who want to enter the Museum *without* booking in advance:

1. Visitors enter a free access area without queuing. Here, on a digital kiosk, they check the availability of tickets for the current day;
2. On the same kiosk, visitors select—among those available—the preferred visiting time;
3. Visitors leave the Museum area. At the chosen time, they come back and can enter the Galleria straightaway.

Our solution owes its success to the ability of accurately predicting the number of visitors present in the Museum at any time of day and, consequently, appropriately modulating ticket availability. To this aim, we need in practice to predict the *daily number of visitors*, their *arrival time* and the *expected duration* of their visit.

### Related works

Tourism forecasting and demand modeling have a long-standing tradition in tourism economics research and several surveys (Jiao and Chen 2018; Li et al. 2005; Wu et al. 2017; Song and Li 2008) have been written over the years, analyzing about 1000 studies published from 1960 to 2018. From this rich literature one can identify three main modeling trends: econometric, time series, and artificial intelligence (AI) models. Although pure and hybrid AI models are nowadays very trendy, also thanks to the availability of data provided by tourists online experience, econometric and time series models remain a consolidated option. In particular, advanced time series models can easily handle trends and seasonality, and the most widely used methods for tourism forecasting are the *Auto Regressive Integrated Moving Average* (ARIMA) models (Shumway and Stoffer 2011). In Sect. 4.1 we describe an ARIMA model able to effectively predict the daily number of visitors at Uffizi.

Prediction of visit duration is strictly related to the visitors’ behavior in the Museum. Seminal studies on visitors’ behavior inside a museum date back to 1928 (Robinson 1928). They have recently received greater attention thanks to new digital technologies and devices [like RFID (Wikipedia 2021; Lanir et al. 2017), Wi-Fi (Georgievska et al. 2019; Hong et al. 2018), Bluetooth (Oosterlinck et al. 2017; Delafontaine et al. 2012; Yoshimura et al. 2014) and video cameras (Ruggiero et al. 2018)] that allow detailed and continuous indoor *visitors tracking* (Gu et al. 2009). A visitor tracking solution measures the *visitors trajectory*, by which rush hours and visit duration can be estimated. Each tracking solution has its trade-offs between cost, practical implementation issues, different visitor engagement, and scale and accuracy of measurements. In Sect. 3 we discuss the simple hardware and software tracking solution that we have implemented at Uffizi.

Several (mathematical) models of visitors’ behavior have also been studied in literature. A general taxonomy (Kuflik et al. 2012; Véron and Levasseur 1989) classifies individual behaviors into four categories: *Ants* (typically follow a specific path and intensively enjoy the artworks); *Butterflies* (do not follow a specific path but are guided by the physical orientation of the exhibits and stop frequently to look for more details); *Fish* (move around in the center of a room and usually avoid looking at artworks details); and *Grasshoppers* (seem to have a specific preference for some selected artworks and spend lot of time on them). Social behavior too has been widely investigated (Dim and Kuflik 2014; Lanir et al. 2017), and visitors are typically classified into the following categories: *Penguins* (walk through the museum but do not pay attention to the exhibits), *Geese* (advance together, with one clear leader), *Meerkats* (advance standing side by side and paying synchronously a lot of attention to the artworks), *Parrots* (share their attention between artworks and other group members, interacting while looking at the exhibits), *Doves* (stand face to face, involved in conversation, ignoring the artworks) and *Lone Wolves* (enter the museum together and then separate).

Clustering and AI techniques have been used to classify visitors into the above categories. Then, starting from this categorization, several predictive models have been devised and some of them guarantee an accurate prediction down to room-level (Delafontaine et al. 2012; Kuflik et al. 2012; Dim and Kuflik 2014; Martella et al. 2017; Yoshimura et al. 2014, 2019; Centorrino et al. 2021). The ability of predicting visitors trajectories is required to create a (realistic) *digital twin* of a museum that can be used to design (by simulation/optimization techniques) new visitors management policies (Centorrino et al. 2021).

The model we discuss in Sect. 4.3 does not require the room-level granularity of the models above. It also works without clustering the visitors. In fact, we show that visit duration at Uffizi follows a gamma distribution whose parameters can be accurately estimated.

## The hardware/software infrastructure for data acquisition and recording

Collecting accurate data in a context like the Uffizi Museum is a demanding task as it requires tackling various practical problems: the absence of a reliable and flexible network infrastructure, the difficulty of installing physical devices in the rooms (there is a high risk of damaging historical artifacts), and also legal issues like the responsibility of data management under the European Union Directive 95/46/EC [*General Data Protection Regulation* (European Parliament 2016)].

An anonymous and non-intrusive technological solution to monitor visitors flow is based on RFID technology (Wikipedia 2021). Ideally speaking, all the necessary information (arrival time, queuing time, entry time and exit time) can be systematically collected by providing each entering visitor with an RFID tag and by installing RFID readers in suitable positions (one near the main entrance, two on the second floor, one at the cafeteria, and two more on the first floor and near the main exit). RFID readers could be positioned on standing panels, thus overcoming the difficulty of installing physical devices in the rooms. Those readers would also provide information on the partial visiting time of specific Museum sections. However this solution would prove expensive, as the cost of an RFID tag being around 0.10 Euro, and the number of visitors up to 8000 per day.

**Fig. 2:**
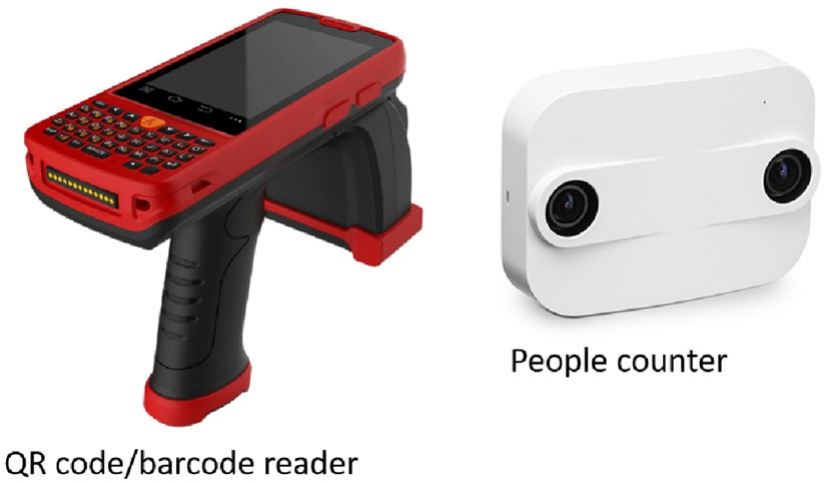
The reader and people counter

This aspect motivated us to leverage alternative and accessible data sources, and our choice fell on the unique anonymous barcode ID carried by each ticket issued at the Uffizi. As the installation of new turnstiles with barcode readers is expensive and complex, we deployed programmable barcode readers (see Fig. 2) operated by human operators at the entry and exit points of the Gallery. The readers acquire the ID printed on each ticket and send the information in real time to a server which runs an application designed to store and clean up the collected data. Given human operators availability, this solution is easily scalable when new monitoring points are added (for instance, during the experimental period, the Museum successfully added a new exit point for short visits, namely the staircase by architect Natalini^1^). On the other hand, being human operated, this solution is error prone. For this reason, we first cross-validated the data by means of RFID tags released to a (small) subset of visitors; we then complemented the data collected by barcode readers with those collected by newly installed people-counter devices (Fig. 2), and also by the images caught via (a subset of the) 500+ CCTV cameras already installed inside and outside the Gallery. We tested different people-counting sw applications to be run on data from the existing cameras and we eventually found a solution that, by the use of legacy cameras, gives precise results.

In compliance with the Uffizi practice, we divided the day into 15-min time slots (choice subsequently validated by statistical analysis) and arranged our final dataset into 37 slots corresponding to standard day opening hours.

**Fig. 3:**
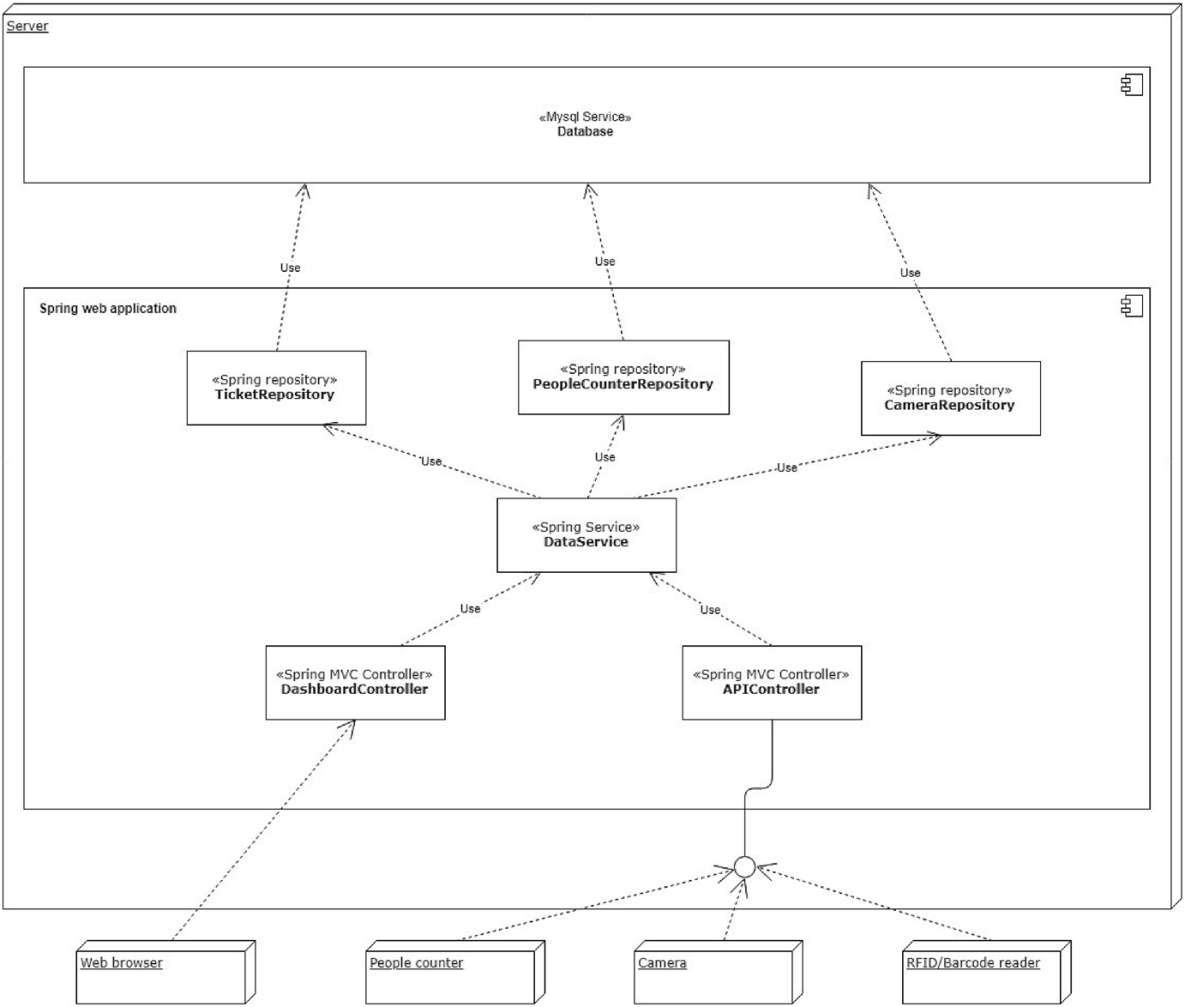
The data acquisition software architecture

Figure 3 shows the architecture of the software we developed for data acquisition. It was designed according to the *Model-View-Controller* (MVC) design pattern (Wikipedia 2021), and implemented using the Spring framework (Pivotal 2019). The application controllers are principally used to set up an interface that exposes a data acquisition service through RESTful API. This is the interface used to send data by the devices described above. Precisely, data acquired by human-operated readers, RFID tags and barcodes come from an ad-hoc developed application, while the data source producing visitors counts and people density in the Gallery rooms relies on device-embedded systems that send information every minute. In addition, another controller allows authorized users to monitor data acquisition, with interactive dashboards offering information about visit time and visitors counts (examples of plots provided by such dashboards are reported in Fig. 13a and b). The data collected are stored into a MySql Database Management System, which also provides procedures to calculate both summary and real-time statistics.

## Data analysis and predictive models

Using the above described infrastructure, we collected data over about two years, until June 2018. We also considered aggregated visitors data provided by the Uffizi starting from 2014. Then, we performed a detailed data exploratory analysis on the following data types:

1. Time series of daily entrances to the Museum from 2014 to June 2018 and, relatively to the last two years period, also the time series of entrances by reserved tickets;
2. Per-visitor entry and exit time, to estimate visit duration. The duration varies greatly depending on entrance time. This survey considered approximately 40 days, with particular emphasis on the first Sundays of each month, which is a free entrance day. Each day opening time was divided into 37 slots of 15 min each, corresponding to the time slots available for ticket booking via web (in previous days).

The analysis aimed at:

1. Providing a daily forecast of the number of visitors;
2. Estimating the expected visit duration as a function of entrance time slots.

For the prediction model, we used ARIMA models (Shumway and Stoffer 2011) with good results.

**Fig. 4:**
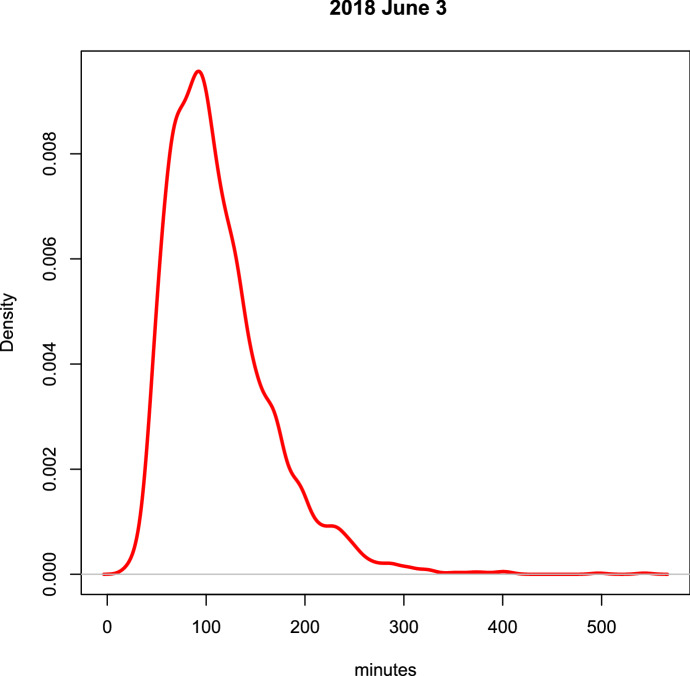
Visiting time on June 03rd, 2018

Although the series proved to be highly volatile due to the impact of concomitant public holidays, the further addition of information related to the time series of the reserved tickets [through a *Transfer Function Analysis* (TF) model of (Box and Jenkins 1970)] considerably improved the prediction accuracy. One particular problem we needed to overcome is the absence of reserved-entrance data for free Sundays, when advance booking is not allowed. Hence, via typical tools of data analysis such as factorial analysis and clustering, we attempted to identify a characteristic week profile week by analysing the weekly cycle, thus obtaining an improved forecast for free-entrance Sundays. As regards the probability density function of visit duration we found out that, with good approximation, it behaves like a gamma-type stochastic variable (by way of example, see the data of the first Sunday of June 2018 shown in Fig. 4).

It is important to emphasize that visit duration, of vital importance in the simulation of behaviors and consequently for booking same day tickets, is variable throughout the day, ranging on average from over 120 min in the early morning to around 100 min in the late morning, and decreasing to an average of 60–70 min for visitors entering after 16:00 h. This type of variability was verified in different periods of the year, summer to winter.

### Prediction of daily visitors number

**Fig. 5:**
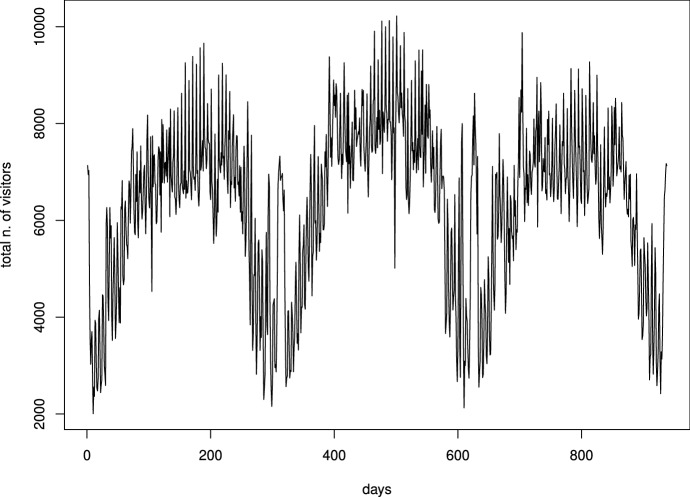
Time series of daily visitors from January 2016 to December 2018 ($Y_t$)

**Fig. 6:**
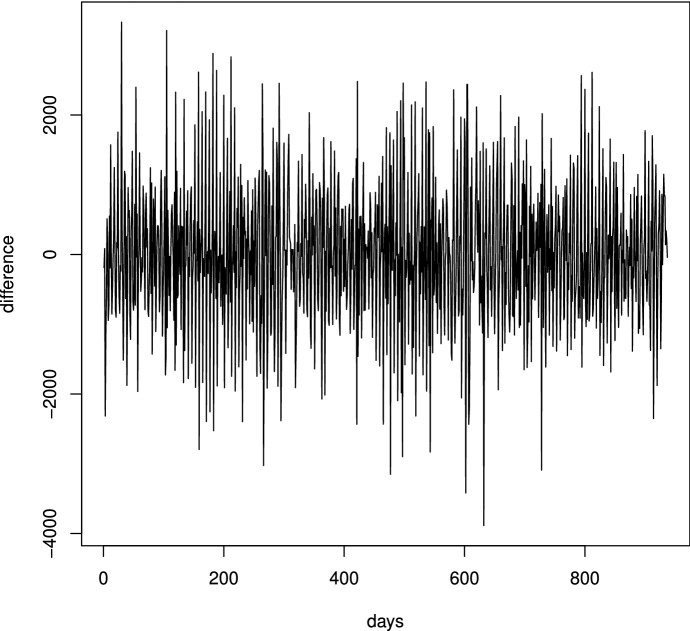
Differentiated time series $Z_t = \nabla Y_t=Y_t-Y_{t-1}$

The time series available consists of the per-day number of visitors. It shows special features, since the Museum closes on Mondays and on other special holidays. Furthermore, such incidents as strikes, particular weather conditions, air conditioning failures, have caused anomalous series variations. This made it necessary to re-organize or modify the data so as to make the series more homogeneous from a statistical point of view. One of the series representations, shown in Fig. 5, is relevant to the period January $2\text {nd}$, 2016 – December $31\text {st}$, 2018. Data seasonality appears evident from the figure (visitors in fact increase in spring and summer), as well as a marked daily volatility. The use of ARIMA modeling requires series stationarity: in simple terms, constant mean and variance. We achieved this feature by taking the raw differences of the series, which resulted in the differentiation presented in Fig. 6.

The high volatility did not allow to obtain satisfactory forecasts. In view of this drawback, we introduced the series of visitors who booked in advance. This new series significantly improved the features of the forecasting model. The ARIMA model that provided excellent results was ARIMA(5, 1, 0):

$$\begin{aligned} Y_t = Y_{t-1}+\varphi _{1}Z_{t-1}+\varphi _{2}Z_{t-2}+\ldots +\varphi _{5}Z_{t-5}+\alpha \; X_t+\epsilon _t, \end{aligned}$$

where

- $Y_t$ is the time series of daily visitors
- $Z_t$ is the differentiated time series
- $\varphi _1,\varphi _2,\ldots \varphi _5$ and $\alpha$ are coefficients estimated by the model
- $X_t$ are the visitors booked for time *t* that are known at time $t-1$
- $\epsilon _t$ is a sequence of independent random variables (white noise)

**Fig. 7:**
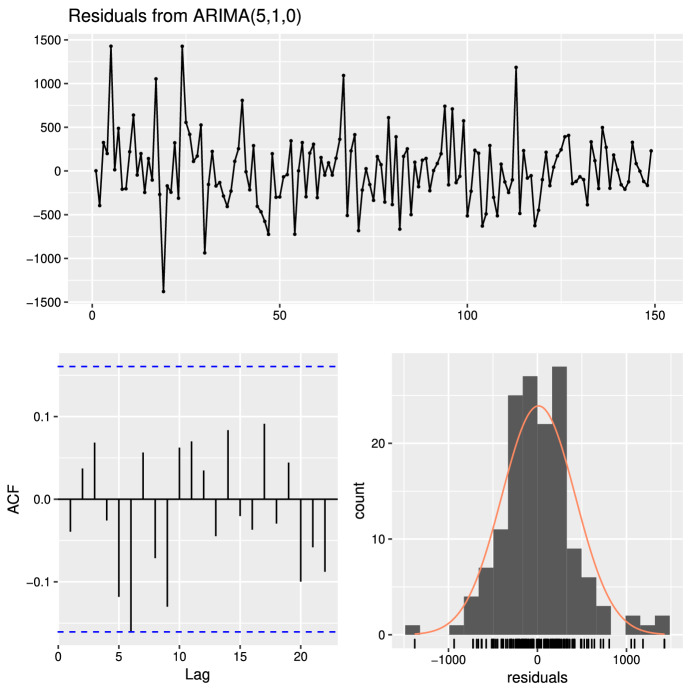
Plot of the ARIMA(5, 1, 0) process

Figure.7 resumes the model output via the analysis of residuals (estimated white noise by model). The figure confirms the validity of the model since, as expected, the spectrum is a white noise. All elaborations were made using the software package R (Team RC 2019).

### Improving the prediction: weekly schedule

A further aid to the forecast is provided by the study of the weekly cycle, that is a data cycle linked to the days of the week, which was found to be independent of visitor numbers. To this end, data were first structured as a matrix, where every row contains data relevant to a week from Tuesday to Sunday (recall that the Museum is closed on Mondays). To highlight a week profile, data were re-cast as a percentage of the total number of visitors per week. Part of this matrix is shown in Table 1.

**Table 1:** Data week profile

	Week	Sun	Tue	Wed	Thu	Fri	Sat
1	04/01/15	28.34	20.26	13.26	11.26	13.18	13.69
2	11/01/15	22.16	15.85	16.44	11.92	13.12	20.52
3	18/01/15	17.86	16.82	14.73	14.54	15.22	20.83
4	25/01/15	20.81	16.18	12.06	15.24	14.58	21.14
5	01/02/15	27.58	15.99	13.26	11.78	13.74	17.64
6	08/02/15	18.40	16.39	12.96	13.03	16.08	23.14
7	15/02/15	20.31	16.12	16.14	15.76	14.02	17.65
8	22/02/15	18.77	17.98	14.54	14.50	14.89	19.32
9	01/03/15	24.23	15.83	15.10	13.79	13.39	17.65
.	.	$\ldots$	$\ldots$	$\ldots$	$\ldots$	$\ldots$	$\ldots$
.	.	$\ldots$	$\ldots$	$\ldots$	$\ldots$	$\ldots$	$\ldots$

**Fig. 8:**
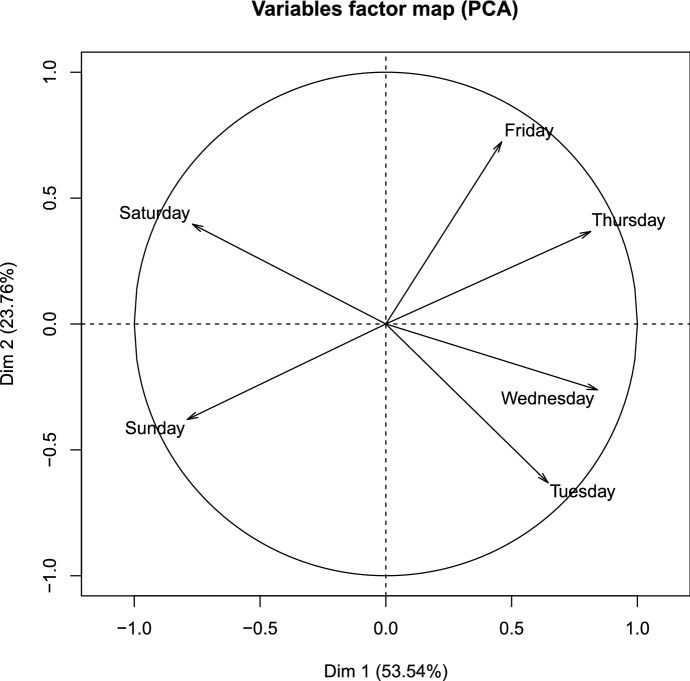
Correlation between the percentage of visitors on different week days

This operation aims at identifying similarities in behaviors, regardless of the particular time of year. Those data were then classified, after reduction with *Principal Components Analysis* (PCA) (Greenacre 2018), in order to eliminate redundancies, to highlight profiles and to enable a graphical representation of those profiles. An interesting result is shown in Fig. 8, which shows the correlation between the percentage of visitors on different week days.

We point out that the classification problem can be considered an open chapter of mathematical statistics, since the answer to the question *how many groups exist of a specific set of data?* hardly ever has a unique and optimal answer. This said, we tackle the problem of identifying a number of groups, based on a fundamental concept known as *the Principle of Parsimony* (Sober 1981). In fact, unless particular endogenous and/or exogenous constraints are involved, to understand and use reference groups it is necessary for them to be limited in number. Complying with this principle, in our case we look for a solution with $\le 3-10$ groups. In particular, we use two classification tools which, with a further dose of common sense, would yield an acceptable solution. It is important to stress that, to facilitate the analysis of the results with the different methodologies employed, the input data were made homogeneous by performing all tests using the matrix of factorial coordinates rather than raw data. This is done in order to use the most stable information, also considering that more than 76% of the total variance is expressed by the first two factorial coordinates.

**Fig. 9:**
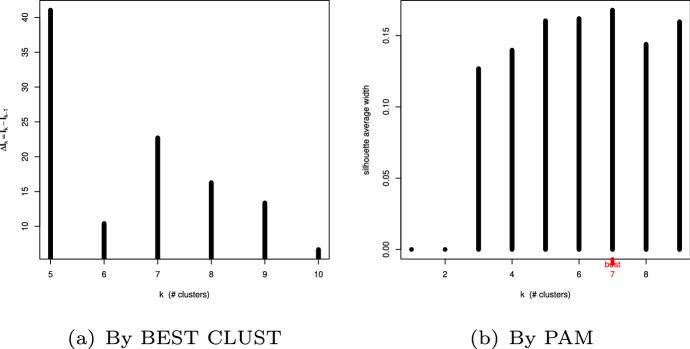
Optimal number of groups

The first tool used, BEST CLUST (Attanasio et al. 2013; Maravalle and Simeone 1995) is based on the decrease of internal inertia, that is the intra-group variance as the number of groups increases. It is related to the Huyghens’ formula that binds the sum of the individual groups’ internal inertia to the total one. Its application shows relative maxima corresponding to 5 or 7 groups, as shown by the leftmost plot of Fig. 9.

The second classification tool used was *Partitioning Around Medoids* (PAM) (Kaufman and Rousseeuw 1990). It also provided a chart that highlights the optimal number of seven groups as shown in Fig. 9, right.

Following this analysis, we decided for a seven group classification.

### Prediction of visit duration

**Fig. 10:**
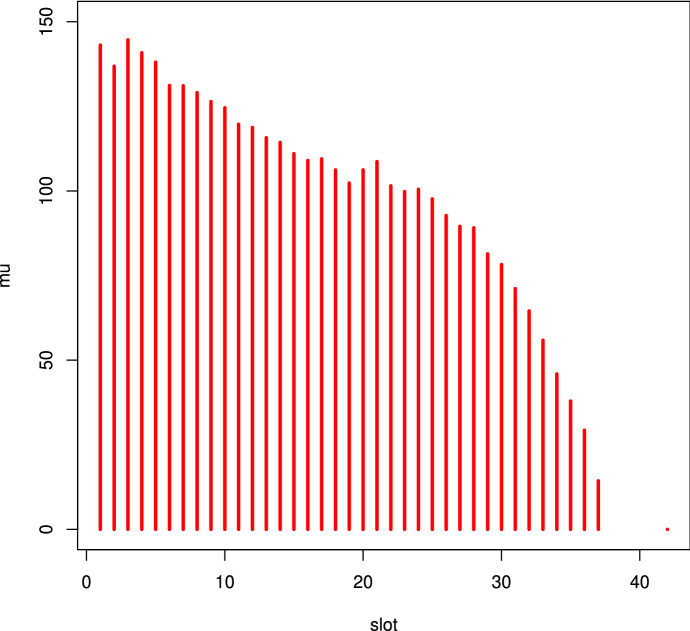
Average duration of the visit as function of entrance slot

A fundamental parameter for queue management is the duration of the visit to the Museum (Centorrino et al. 2021). As one can easily understand, the visit duration is a function of many subjective parameters and, there fore, varies significantly from subject to subject. Figure 10 presents a plot of the average duration as a function of entrance slot. Visit time shows a decreasing (but not linear) trend during the day, that is: the earlier the visit the longer the duration. This behavior occurs regardless of the day of the week or the time of year.

A probabilistic trend was then highlighted within each slot, strongly characterized and linked to the gamma distribution shown in Fig. 4, whose analytical expression is:

$$\begin{aligned} f(x,r,\lambda )=\frac{\lambda ^r\;x^{r-1}}{\Gamma (r)}\;e^{-\lambda \;x}\ \ \ \ \ \ \ x>0 \end{aligned}$$

where

- *x* is the random duration
- $\Gamma (r)$ is the mathematical function gamma
- $\lambda$ and *r* are characteristics linked to the duration mean $\mu _{X}$ and variance $\sigma ^2$ by the relationship:

$$\begin{aligned} r=\left( \frac{\mu _{x}}{\sigma }\right) ^2\ \ \ \ \text {and}\ \ \ \ \lambda =\frac{\mu _{x}}{\sigma ^2} \end{aligned}$$

We could therefore characterize the trend by associating two numbers with each entry slot: the average $\mu$ and the standard deviation $\sigma$. Analyzing the data collected with daily surveys between the end of 2016 and the beginning of 2017—for a total of 65,536 admissions—we obtained the following Table 2, where:

- *slot* is the entrance time slot
- *num* is the number of visitors in the time slot of the entire period
- $\mu$ and $\sigma$ are the mean and the standard deviation of the visit duration

**Table 2:** Average and standard deviation of visit duration as a function of the entrance slot

	Slot	Num	$\mu$	$\sigma$		Slot	Num	$\mu$	$\sigma$
1	8:15–8:30	492	143.09	59.13	20	13:00–13:15	1879	106.24	43.55
2	8:30–8:45	1606	136.89	54.51	21	13:15–13:30	1505	108.68	45.09
3	8:45–9:00	2047	144.67	58.11	22	13:30–13:45	1465	101.52	41.08
4	9:00–9:15	2172	140.83	58.78	23	13:45–14:00	1564	99.81	38.07
5	9:15–9:30	2392	138.07	58.33	24	14:00–14:15	2052	100.49	38.26
6	9:30–9:45	2625	131.16	58.82	25	14:15–14:30	1746	97.66	34.15
7	9:45–10:00	2853	131.09	55.74	26	14:30–14:45	1513	92.73	34.93
8	10:00–10:15	3258	129.06	55.44	27	14:45:15:00	1413	89.56	30.72
9	10:15–10:30	3038	126.40	52.06	28	15:00–15:15	1339	89.12	28.51
10	10:30–10:45	2957	124.59	54.17	29	15:15–15:30	1246	81.41	24.64
11	10:45:11:00	2842	119.70	51.33	30	15:30–15:45	1046	78.25	24.16
12	11:00–11:15	2708	118.77	52.45	31	15:45–16:00	835	71.16	20.59
13	11:15–11:30	3224	115.73	49.92	32	16:00–16:15	551	64.56	16.26
14	11:30–11:45	2691	114.36	47.76	33	16:15–16:30	320	55.91	16.49
15	11:45–12:00	2546	111.04	48.43	34	16:30–16:45	135	45.95	12.99
16	12:00–12:15	2711	109.02	48.16	35	16:45:17:00	111	37.92	10.03
17	12:15–12:30	2435	109.49	47.91	36	17:00–17:15	11	29.31	13.66
18	12:30–12:45	1925	106.23	46.64	37	17:15–17:30	1	14.35	$-$
19	12:45:13:00	2282	102.35	46.90					

**Fig. 11:**
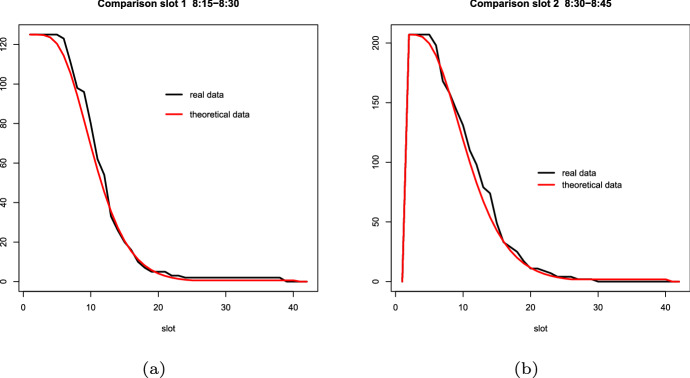
Comparison of live data and model predictions for the number of visitors leaving the premises as a function of exit time for two different entrance slot on Sunday, June 3rd, 2018

At this point, for each slot *t*, we calculated an output probability vector ${\mathbf{p}}_t$, based on the characteristic gamma function of that slot. Each component $p_h$ of the vector represents the probability that a visitor entering the Museum at slot *t* leaves the museum at slot *h*. Trivially, $p_h=0$ if $h \le t$ and the sum of all $p_h$ is equal to one. The collection of vectors for each *t* form a matrix that we refer to as *Output Probability Matrix* $\mathcal P$, whereby it is possible to evaluate the exit flow for a given visitor entry flow. This allows us to dynamically estimate the number of people inside the Museum, and to determine the maximum number of visitors for each entry slot without exceeding the Museum capacity. Live data and model predictions are compared in Fig. 11, showing the number of visitors leaving the premises as a function of exit time, for two different entrance slots on Sunday, June 3rd, 2018. The agreement is excellent.

An important feature, which lends general validity to the model devised in the present study, is the evaluation of the daily number of visitors as a function of the Museum capacity. The latter then becomes a simple parameter which can easily be tailored to the specific requirements of different buildings with controlled access.

## Prescriptive model

Given the output probability matrix $\mathcal P$ returned by the prediction model, we want to calculate the number of tickets that can be issued for each slot *t*. The model must consider:

1. The capacity of the x-ray body scanners in the main entrance, equal to $S_t$ visitors per time slot;
2. The maximum number of allowed visitors in the exhibit area, $C_t$;
3. The ticket price, $u_t$;
4. The number of tickets reserved (either in the same day or in previous days), $A_t$.

The goal is to maximize the Museum income without introducing congestion (delays) at the entrance. The model uses the following (integer) variables:

$$\begin{aligned} x_t = \text {number of tickets issued for slot } t \end{aligned}$$

The objective function amounts to maximize the Museum total income, that is:

$$\begin{aligned} \max \sum _{t \in \mathcal T} u_t x_t \end{aligned}$$

In what follows we discuss results for free-entrance days, in which the Museum wants to maximize the number of visitors (corresponding to $u_t=1$ for all slots). The system, for each time slot *t*, has the following constraints:

- (i) the maximum capacity $S_t$ of the x-ray body scanners in the main entrance represents a bound on the maximum number of tickets issued for slot *t*:
  $$\begin{aligned} x_t \le S_t \;\;\;\; t \in \mathcal{T} \end{aligned}$$
- (ii) the maximum number of allowed visitors in the exhibit area, $C_t$, cannot be exceeded.

To implement the latter constraint, we need to evaluate the number of visitors present in the Museum in each time slot *t*. Under the assumption that the visitors entering during slot *t* exactly correspond to the tickets issued in that slot, the number of visitors in slot *t* are equal to the number of tickets issued in slots $\{1, \ldots , t\}$: that is, $\sum _{h = 1}^{t} x_h$ minus the number of visitors who left in slots $\{1, \ldots , t-1\}$. The latter figure can be evaluated from the output probability matrix $\mathcal P$, being $p_{tj} x_t$ the expected number of visitors entering the Museum at *t* and leaving it at *j*. The resulting linear constraint has the form:

$$\begin{aligned} \sum _{h = 1}^{t} x_h - \sum _{h=1}^{t-1} \sum _{j=1}^{t-1} p_{hj} x_h \le C_t \end{aligned}$$

and the complete model reads:

1 $$\begin{aligned}&\max \sum _{t \in \mathcal T} u_t x_t \end{aligned}$$

2 $$\begin{aligned}&\sum _{h = 1}^{t} x_h - \sum _{h=1}^{t-1} \sum _{j=1}^{t-1} p_{hj} x_h \le C_t&t \in \mathcal{T} \end{aligned}$$

3 $$\begin{aligned}&A_t \le x_t \le S_t&t \in \mathcal{T}\nonumber \\&\qquad {\mathbf{x}} \in \mathbb {Z}^\mathcal{T} \end{aligned}$$

Note that in (3) we introduced a lower bound on tickets availability, that accounts for the tickets to be issued in *t*. Two facts determinate $A_t>0$: the first concerns the tickets reserved in advance (i.e., in days preceding the current planning day); the second concerns model execution at time $\bar{t}>1$, when tickets for time slots $t < \bar{t}$ have already been issued.

All in all, the ILP model (1)–(3) contains $\vert \mathcal{T} \vert$ integer variables and $\vert \mathcal{T} \vert$ constraints and it is easily solvable by Commercial-Off-The-Shelf ILP solvers (CPU time typically does not exceed seconds). This is a crucial property, since the model is repeatedly solved during the opening hours of the Museum.

**Fig. 12:**
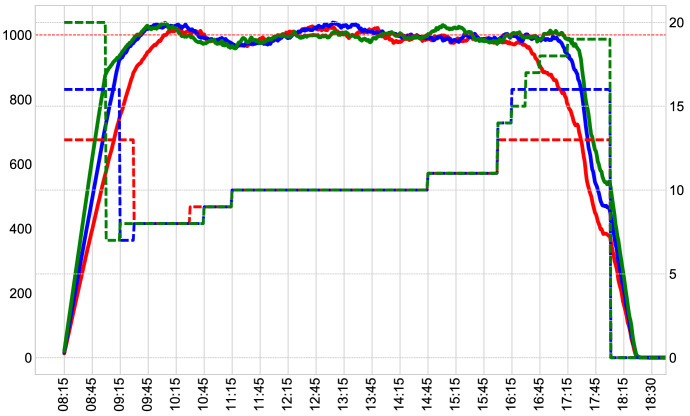
Simulation with $C_t=1000$, $A_t=50$ and $S_t=\{200, 250, 300\}$

An example of the system dynamics is reported in Fig. 12. The graphs are obtained by evaluating optimal solutions ${\mathbf{x}}^*$ to model (1)–(3) at the beginning of the day, with the matrix $\mathcal P$ defined as in Sect. 4 and with $u_t=1$, $A_t=50$, $C_t=1000$ and $S_t=\{200, 250, 300\}$ (respectively, red, blue and green lines in the chart). Then, we simulated the system for each time slot *t* assuming that all $x^*_t$ visitors enter the Museum at slot *t*, flowing at regular pace through the x-ray scanners. Each visitor will stay in the system for a time drawn from the $\gamma _t$ distribution associated with *t*. Dashed lines in Fig. 12 represent the number of visitors per minute entering the Museum (values are reported on the right *y*-axis). Continuous lines represent, with a granularity of one minute, the number of visitors present in the exhibit area (values are reported in the left *y*-axis). The three scenarios lead to 6400, 6750 and 7050 total visitors respectively. In this simulation one can observe that the capacity constraints (2) are substantially fulfilled for the whole time horizon, despite the approximations introduced by the 15-min time slot discretization and the roundoff error introduced in representing the coefficients of $\mathcal P$ in model (1)–(3). In other worids, for all scenarios and after the initial ramp-up, the number of visitors in the system fluctuates around 1000. Recall that in the real system, a visitor cannot enter the exhibit area if more than $C_t$ people are already in. Thus, when $C_t$ is exceeded, a queue (i.e., a delay from the estimated entering time) occurs. These periods appear rather limited in all three scenarios.

Scenarios mainly differ by the time in which the exhibit area is filled up. Of course, a rate of 300 people per time slot results in an overall larger number of visitors, but such a rate requires structural changes in the visitor body scan procedure. Interestingly, a longer time to saturate the system may be useful for the decision maker to correctly assess the system dynamics. In fact, during Museum opening hours, model (1)–(3) is run every 15 min (at the end of each time slot) and it is updated with real time data. If $\bar{t}$ is the time slot at which the model is executed, the following changes affect model (1)–(3):

1. $x_t$ variables for $t < \bar{t}$ are fixed at a value corresponding to the number of visitors actually entering the Museum at *t*;
2. Tickets that have already been issued for slots $t \ge \bar{t}$ must be considered, and $A_t$ updated accordingly;
3. After the first hour (i.e., for $t > 4$) one can also check the correctness of the distributions used to predict visitors behavior, as a statistically significant number of visitor has already left the Museum. In case of a noteworthy deviance, one can also change matrix $\mathcal P$ for $t \ge \bar{t}$;
4. $C_t$ can be updated as well.

In practice, having the system saturated later in the day (as in the scenarios with $S_t=\{200, 250\}$) leaves more room for assessing visitors behavior. Furthermore, by running the model and simulating the system every 15 min during the day, one can visualize the real and estimated complete day trend and make decisions accordingly.

**Fig. 13:**
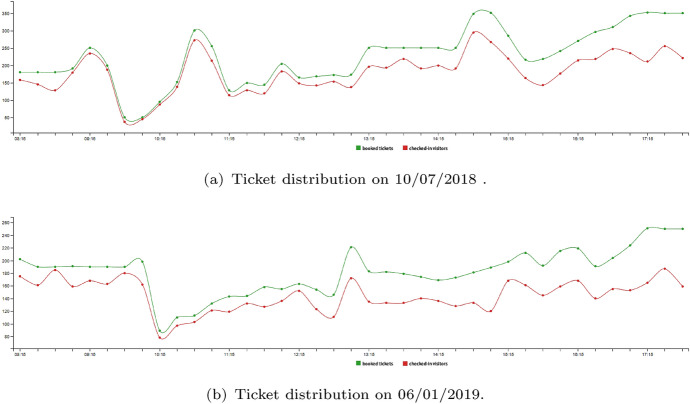
Ticket distribution

Figure 13a and b report an example of on-field results. Graphs show the realized tickets distribution on two free-entrance days (10/07/2018 and 01/06/2019). The green line represents the tickets issued, the red one the checked-in visitors in each time slot.

First, one observes that the number of issued tickets significantly varies over the day w.r.t. the ideal simulation of Fig. 12. However, this does not affect the time visitors physically spend queueing, since the difference between the expected and the actual entry time for both days never exceeded 17 min (with an average of 14 min). This basically means that almost all visitors actually enter the Museum in their booked slot.

From the graph one can also observe that the difference between the number of tickets issued and the checked-in visitors increases during the day. A detailed analysis of the data shows that reservations in the morning for the afternoon tend to have a higher no-show rate. In our practice, tickets issued but not used are removed from the model, i.e., they are made available starting from the first time slot following the no-show. However, in case of high ticket request (as on free-entrance days), a significantly high no-show rate may result in no tickets availability for long time during the central hours of the day. In this case one can decide to overbook some afternoon slots. The overbooking can be handled through parameter $C_t$. Basically, one *virtually* increases the Museum capacity by making more tickets available for slots in which the no-show rate is expected to be high. The overbooking level is then chosen by simulating system dynamics for a set of $C_t$ values, and so evaluating the maximum acceptable delay that congestion causes in visit time, if the no-show rate drops to a physiological value.

## The end user hardware/software solution

The combined use of the predictive model and of the prescriptive (optimization) model provides a reliable knowledge of the number of visitors that can access the Museum in a given time slot with little/no waiting time at the Museum entrance. Moreover, at a given time $\bar{t}$, one can calculate the number of available tickets for all the time slots following $\bar{t}$. Thus, our solution is designed to show visitors the next possible entry times, allowing them to choose one, and to print a voucher containing a reservation for a time $t > \bar{t}$.

The solution is deployed through the installation of seven digital kiosks outside the gallery. Each kiosk allows visitors to self-print the voucher with their own specified entry time. Once reserved the voucher, visitors can leave the Uffizi area and enjoy the surroundings until their reservation time. In the near future a free mobile application with the same capability as the digital kiosks will be developed and delivered to the app stores, allowing users to book the visit from a personal device.

By using the same barcode readers described in the previous section, we also developed an application that simply reads the QR code printed on the voucher and checks that the visitor enters in the correct time slot. To keep visitors informed about the current entry time (that is, of possible delays), we placed two big displays outside the gallery which run an application connected to the system that also gives information about the overall voucher availability for the day.

**Fig. 14:**
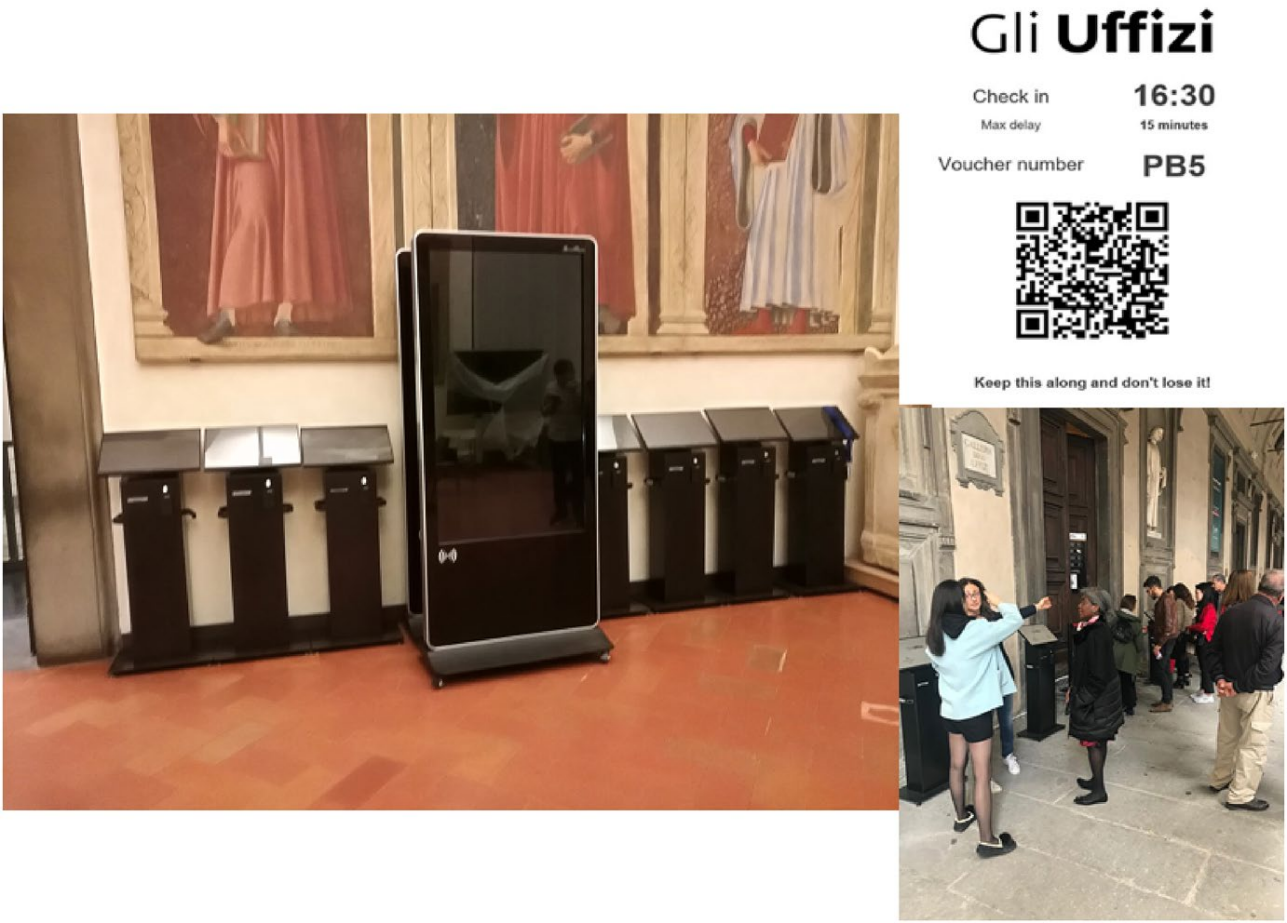
The digital kiosks and monitors in operation

A view of the the digital kiosks infrastructure, displays, and vouchers is shown in Fig. 14. Our system has been implemented on the field during the free-access Sundays since October 2018.

To realize the software components for our solution we developed another Spring Web Application (Pivotal 2019) completed by other external services.

There are three main controllers that expose API to handle vouchers through a unique service: one requests the first available entry time from kiosks; another shows on the big displays general information like the current entry time or the voucher availability at kiosks; the third one gathers information on specific vouchers scanned by the readers at the entrance, and eventually validates them.

Other important tasks are performed by the Scheduler which, where necessary, changes some of the settings to update the optimization results. To achieve this, it uses two services: a simulation service makes use of the probability matrix returned by the prediction model to simulate how many people enter/exit the Museum. This makes it possible to forecast presences inside the gallery in a specific time. The second service, instead, is responsible for calling the external optimization module, represented by a web service developed in Python, and focused on ILP solver functions that are useful to define and execute our model with good performances.

**Fig. 15:**
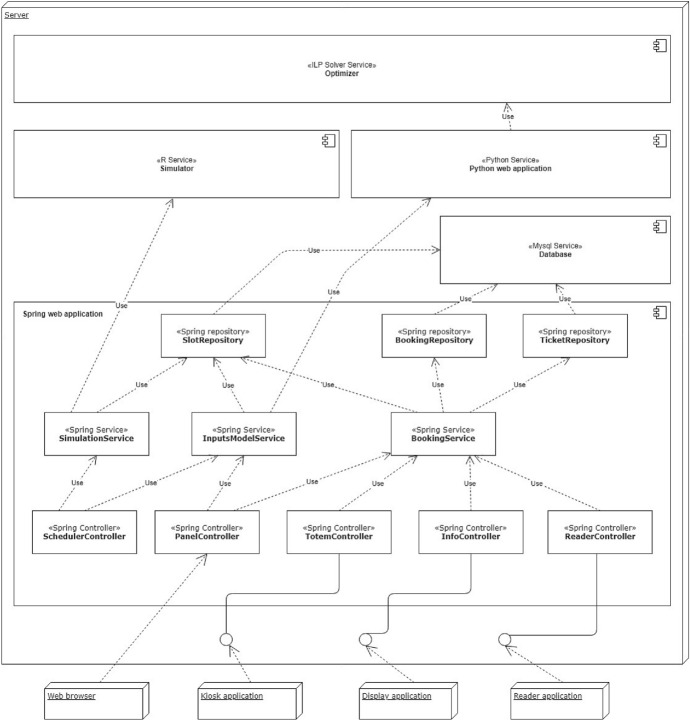
The end-user application software architecture

Results obtained from both calculations or voucher request are stored in a MySQL database (Fig. 15).

## Lessons learned

Looking at the overall experience, we can share the following main lessons learned while designing a data-driven application for monitoring and forecasting visitors of a museum:

- *Infrastructure.* Understanding the data to be acquired goes hand by hand with analyzing the existing infrastructure. The main challenges we faced are: lack of a public WiFi connection inside and outside the Museum, lack of proper turnstiles, legacy cctv cameras provided by different vendors and ranging from very old to very new, limited server infrastructure, lack of digital tickets. Moreover, the historical nature of the building added a number of important constraints, such as: inability to drill walls for attaching new devices, extremely high ceilings that made the use of some people-counting technology unfeasible, limited space to mount proper turnstiles.
- *Human vs. automated data collection.* Human or automated data collection may bring different kinds of challenges. Data collected by human operators may be incomplete due to the difficulty of tracking each single visitor. On the other hand, automated data collection by hardware devices requires extensive software and hardware testing to avoid logging mistakes.
- *How much data is enough?* As above said, we collected data to forecast both the number of visitors and their expected visit duration. One of our concerns was the amount of data sufficient for an accurate forecasting. In order to answer this question, we first refined the daily forecast model so as to reach a discrepancy not larger than 10$\%$ in terms of estimated number of visitors versus the real data. Once achieved this threshold, we compared the estimated visit duration with the one collected during the day. On September 2nd 2018, we ran an in-the-field experiment in Uffizi, to compare at run-time the real visit duration with the estimated one, getting the results discussed in Fig. 11.
- *Booking.* Knowing the number of booked visitors maximizes the precision of our estimate. We discovered that the information about booked visitors increases accuracy to 0.93. The reason is that guided tours and schools are a large share of visitors in the high season and their visits are always booked in advance.
- *External waiting time recording*. The RFID/barcode solution adopted in our approach, although precise and flexible, is quite expensive in terms of human resources (two people required) and hardware (two readers and throwaway RFID tags). We are currently moving towards the use of digital cameras and AI to count crowds and people in lines.

## Conclusions and future work

This article relates our 2-years experience within the UFFIZI Gallery project. By following the process reported in Fig. 1, we identified the data to be collected, created an infrastructure to collect, clean, and merge the data, realized the models, and implemented, deployed and operated the software.

**Fig. 16:**
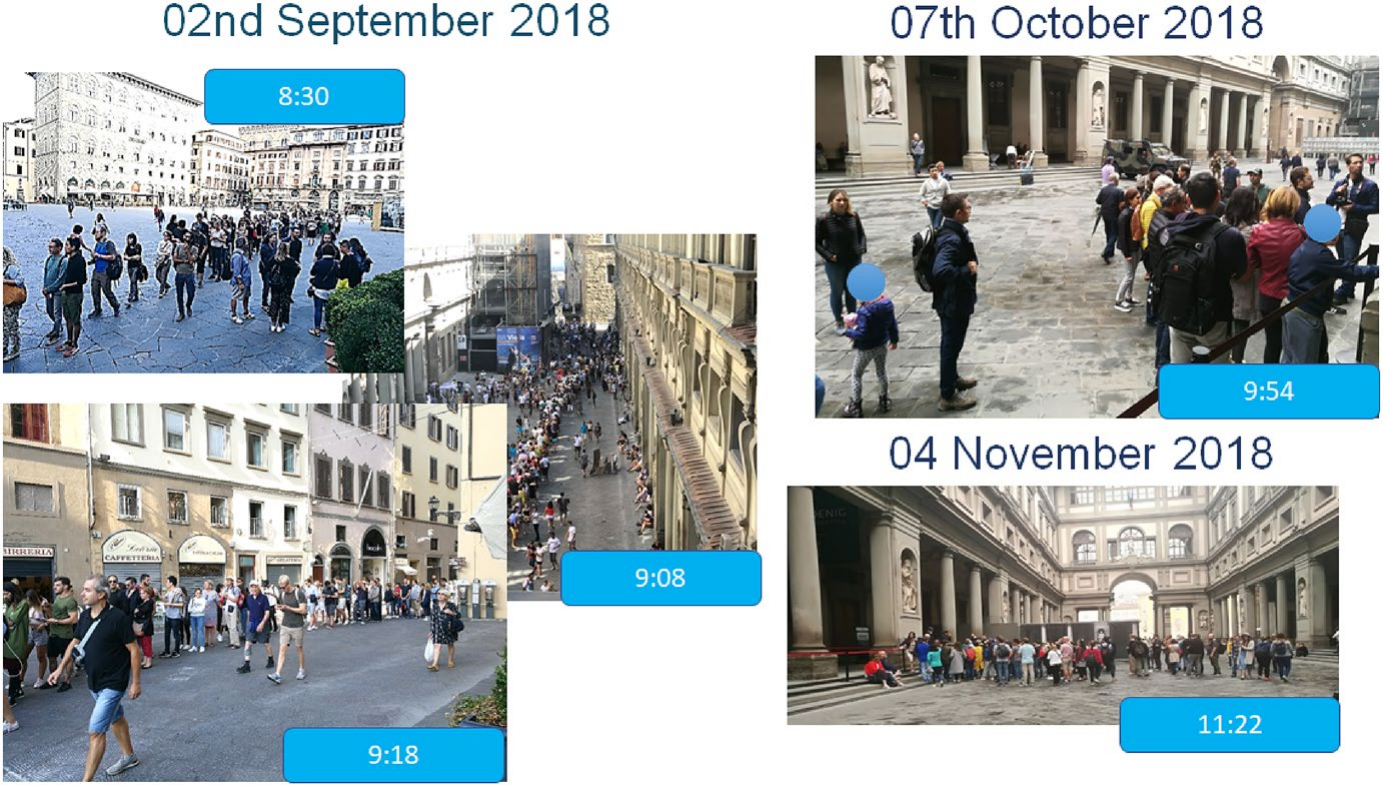
Before and after

Figure 16 provides a visual comparison between the queue observed on September 02nd 2018 (traditional system) and the (almost null) ones on October 07th 2018 and November 04th (our system). We hold the comparison fair, since:

- 6961 people accessed the Museum on September 02nd versus 7765 on October 07th (essentially, we handled more people);
- 7036 vouchers had been distributed at 6:35 pm on September 02nd versus 7385 at noon on October 07th (essentially, the flow of incoming visitors was much higher in October);
- the average queuing time dramatically dropped from 64 min on September 02nd (for a total of 445,504 queueing minutes) to 14 min on October 07th.

In our view, this is the first step towards a longer-term plan.

First of all, cultural tourism is steadily growing, both in Italy and in other EU countries (European Commission 2018), and so is the number of visitors that the most attractive museums and cultural areas are able to attract. Therefore, urban security issues and waiting queues are expected to increase, unless solutions like ours are experimented and developed. Our solution is a valuable approach to a problem which is supposedly growing. We expect and aim to generalize it, with applications to other museums and cultural areas.

Secondly, our approach is currently being extended for improving tourism sustainability. In fact, the time saved from queuing in a museum, can be used to visit other places. This would increase the number of visitors to less known museums located near the main attractions. We developed the preliminary version of a planner that provides information on nearby museums (associated to the Uffizi Gallery) to be visited when the Uffizi is busy. A preliminary empirical analysis, performed immediately before the COVID-19 pandemic broke out, showed that the number of visitors to locations around the Uffizi (for example, Palazzo Pitti) actually increased during the implementation of our system. This can mitigate the consequences of overtourism in the main attractions, at the same time addressing more visitors to nearby museums (Phi 2019).

Thirdly, we plan to add a dynamic pricing strategy so as to change the Museum admission price into one based on supply-and-demand, and on other external market factors.

## Footnotes

1 https://www.uffizi.it/en/notices/apre-lo-scalone-natalini-come-seconda-uscita-degli-uffizi.

## References

[CR2] Attanasio A, Maravalle M, Scalzini A (2013). Different criteria for the optimal number of clusters and selection variables with R. J Math Syst Sci.

[CR3] Box G, Jenkins G (1970). Time series analysis, forecasting and control.

[CR4] Centorrino P, Corbetta A, Cristiani E, Onofri E (2021). Managing crowded museums: visitors flow measurement, analysis, modeling, and optimization. J Comput Sci.

[CR5] Delafontaine M, Versichele M, Neutens T, Van de Weghe N (2012). Analysing spatiotemporal sequences in bluetooth tracking data. Appl Geogr.

[CR6] Dim E, Kuflik T (2014). Automatic detection of social behavior of museum visitor pairs. ACM Trans Interact Intell Syst (TiiS).

[CR7] Egresi I (2018) “tourists go home!”—tourism overcrowding and “tourismophobia” in European cities (can tourists and residents still co-habitate in the city?)

[CR8] European Commission (2018) Cultural Tourism. European Commission. http://ec.europa.eu/growth/sectors/tourism/offer/cultural_en

[CR9] European Parliament (2016) General Data Protection Regulation text. EUR-Lex. https://eur-lex.europa.eu/eli/reg/2016/679/oj

[CR10] Falk JH (2009). Identity and the museum visitor experience.

[CR11] Georgievska S, Rutten P, Amoraal J, Ranguelova E, Bakhshi R, de Vries BL, Lees M, Klous S (2019). Detecting high indoor crowd density with Wi-fi localization: a statistical mechanics approach. J Big Data.

[CR12] Greenacre M (2018). Compositional data analysis in practice.

[CR13] Gu Y, Lo A, Niemegeers I (2009). A survey of indoor positioning systems for wireless personal networks. IEEE Commun Surv Tutor.

[CR14] Hong H, De Silva GD, Chan MC (2018). Crowdprobe: non-invasive crowd monitoring with Wi-fi probe. Proc ACM Interact Mob Wearable Ubiquitous Technol.

[CR15] ISTAT (2016) Istat report on Tourism. ISTAT. https://www.istat.it/it/files/2016/12/Report-Musei.pdf

[CR16] Jiao X, Chen J (2018). Tourism forecasting: a review of methodological developments over the last decade. Tour Econ.

[CR17] Kaufman L, Rousseeuw PJ (1990). An introduction to cluster analysis.

[CR18] Kuflik T, Boger Z, Zancanaro M, Krüger A, Kuflik T (2012). Analysis and prediction of museum visitors’ behavioral pattern types.

[CR19] Lanir J, Kuflik T, Sheidin J, Yavin N, Leiderman K, Segal M (2017). Visualizing museum visitors’ behavior: Where do they go and what do they do there?. Pers Ubiquit Comput.

[CR20] Li G, Song H, Witt SF (2005). Recent developments in econometric modeling and forecasting. J Travel Res.

[CR21] Maravalle M, Simeone B (1995). A spanning tree heuristic for regional clustering. Commun Stat.

[CR22] Martella C, Miraglia A, Frost J, Cattani M, van Steen M (2017). Visualizing, clustering, and predicting the behavior of museum visitors. Pervasive Mob Comput.

[CR23] Oosterlinck D, Benoit DF, Baecke P, Van de Weghe N (2017). Bluetooth tracking of humans in an indoor environment: an application to shopping mall visits. Appl Geogr.

[CR1] ‘Overtourism;? – Understanding and Managing Urban Tourism Growth Beyond Perceptions, Executive Summary. World Tourism Organization (UNWTO); Centre of Expertise Leisure, Tourism & Hospitality; NHTV Breda University of Applied Sciences; and NHL Stenden University of Applied Sciences., Madrid (2018). 10.18111/9789284420070

[CR24] Pechlaner H, Innerhofer E, Erschbamer G (2019). Overtourism: tourism management and solutions.

[CR25] Phi G (2019). Framing overtourism: a critical news media analysis. Curr Issue Tour.

[CR26] Pivotal (2019) Spring: an application framework and inversion of control container for the Java platform. Pivotal. https://spring.io/

[CR27] Robinson ES (1928). The behavior of the museum visitor.

[CR28] Ruggiero L, Charitha D, Xiang S, Lucia B (2018). Investigating pedestrian navigation in indoor open space environments using big data. Appl Math Model.

[CR29] Shumway RH, Stoffer DS (2011). Time series analysis and its applications.

[CR30] Sober E (1981). The principle of parsimony. Br J Phillos Sci.

[CR31] Song H, Li G (2008). Tourism demand modelling and forecasting—a review of recent research. Tour Manage.

[CR32] Team RC (2019). R: a language and environment for statistical computing.

[CR33] Véron E, Levasseur M (1989) Ethnographie de L’exposition: L’espace. Le Corps et Le Sens. Bibliothèque publique d’information du Centre Pompidou, Paris

[CR34] Wikipedia (2021) Model–view–controller. https://en.wikipedia.org/wiki/Model-view-controller

[CR35] Wikipedia (2021) Radio-frequency identification. [Online; last visit 09/28/2021]. https://en.wikipedia.org/wiki/Radio-frequency_identification

[CR36] Wu DC, Song H, Shen S (2017) New developments in tourism and hotel demand modeling and forecasting international journal of contemporary hospitality management. New developments in tourism and hotel demand modeling and forecasting 507–529

[CR37] Yoshimura Y, Sobolevsky S, Ratti C, Girardin F, Carrascal JP, Blat J, Sinatra R (2014). An analysis of visitors’ behavior in the louvre museum: a study using bluetooth data. Environ Plann B Plann Des.

[CR38] Yoshimura Y, Sinatra R, Krebs A, Ratti C (2019) Analysis of visitors’ mobility patterns through random walk in the louvre museum. J Ambient Intell Human Comput 1–16

